# Supplementary material for: Coenzyme A binding sites induce proximal acylation across protein families
Source: Sci Rep. 2023 Mar 28;13:5029. doi: 10.1038/s41598-023-31900-5 (PMC10050154; doi:10.1038/s41598-023-31900-5)

Supplementary Information

Coenzyme A binding sites induce proximal acylation across protein families

Chris Carrico[1#], Andrew Cruz[1#], Marius Walter[1,2], Jesse Meyer[1], Cameron Wehrfritz[1], Samah Shah[1], Lei Wei[1], Birgit Schilling[1], Eric Verdin[1*]

This supplementary information file includes:

- Supplementary Figure 1
- Legend of Supplementary Table 1
- Raw western blot images for figures 5B and 5C

Supplementary Table 1: CoA-Binding Proteins in the Human and Mouse Proteomes

Supplementary Figure 1

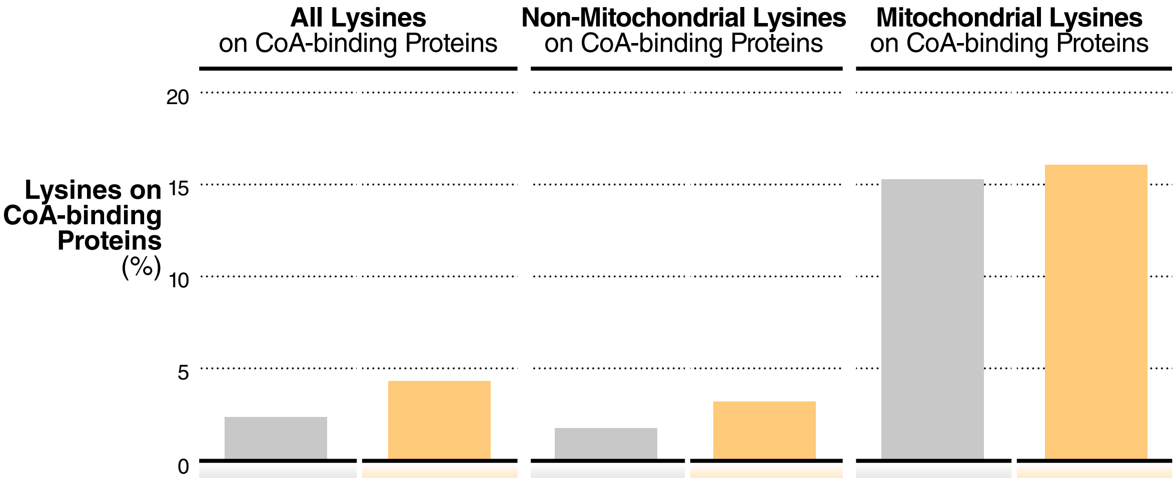

	All Lysines on CoA-binding Proteins		Non-Mitochondrial Lysines on CoA-binding Proteins		Mitochondrial Lysines on CoA-binding Proteins	
	All Lysines	Acetylated Lysines	All Lysines	Acetylated Lysines	All Lysines	Acetylated Lysines
**CoA-binding Proteins**	**15662**	**842**	**11685**	**507**	**3977**	**335**
**All Proteins**	**651194**	**18565**	**624799**	**16575**	**26395**	**2080**
**% CoA Binders**	**2.4**	**4.5**	**1.9**	**3.1**	**15.1**	**16.1**
**Odds Ratio**	**—**	**1.92**	**—**	**1.66**	**—**	**1.08**
***P*-value**	**—**	**3.61E–61**	**—**	**2.00E–24**	**—**	**2.04E–01**

Supplementary Figure 1: Enrichment of acetylated lysines on CoABPs analyzed in a whole-cell human acetylome dataset. Table showing the enrichment of acetylated lysines on CoABPs analyzed in a whole-cell acetylome dataset in human Hela cells. Statistical significances were calculated using Fisher's exact test.

Source Data
Western Blot raw images
Figure 5B

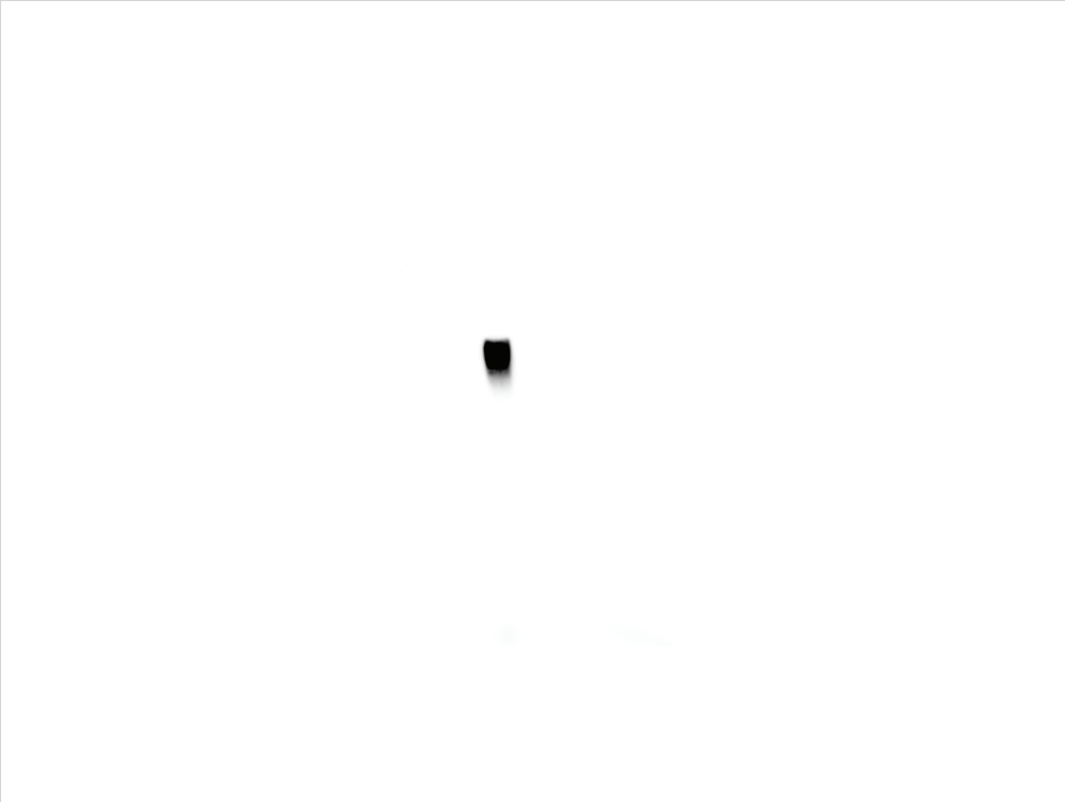

Source Data
Western Blot raw images
Figure 5C

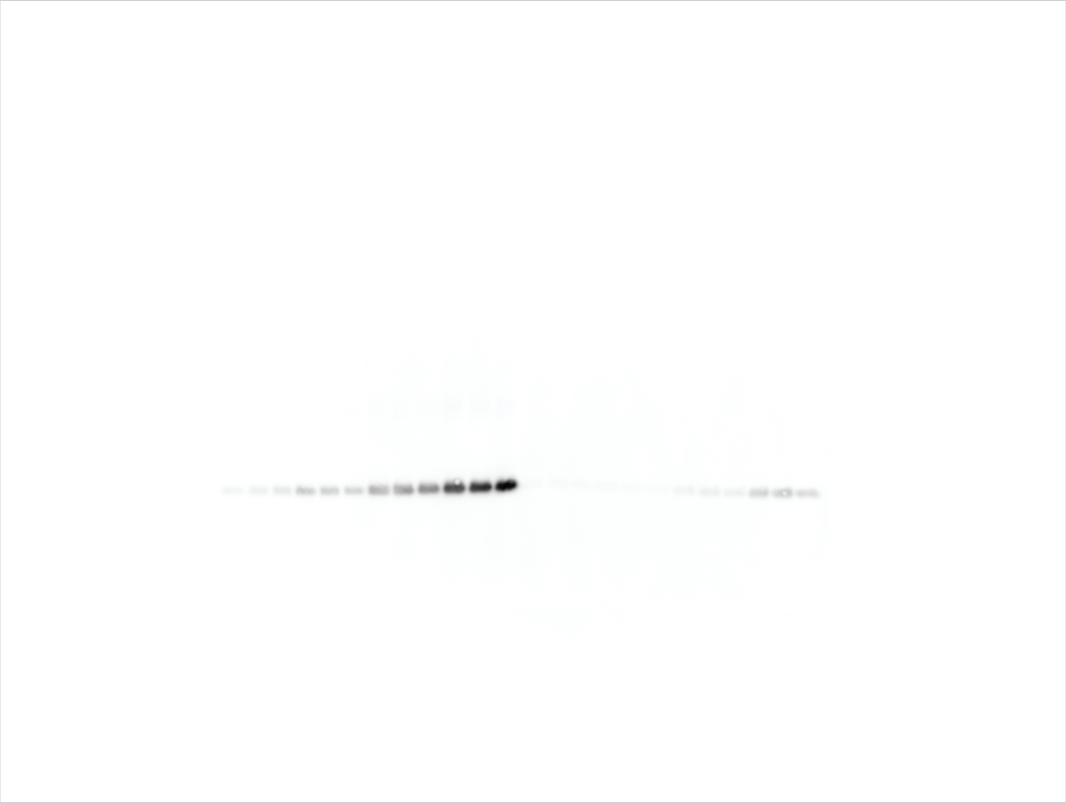

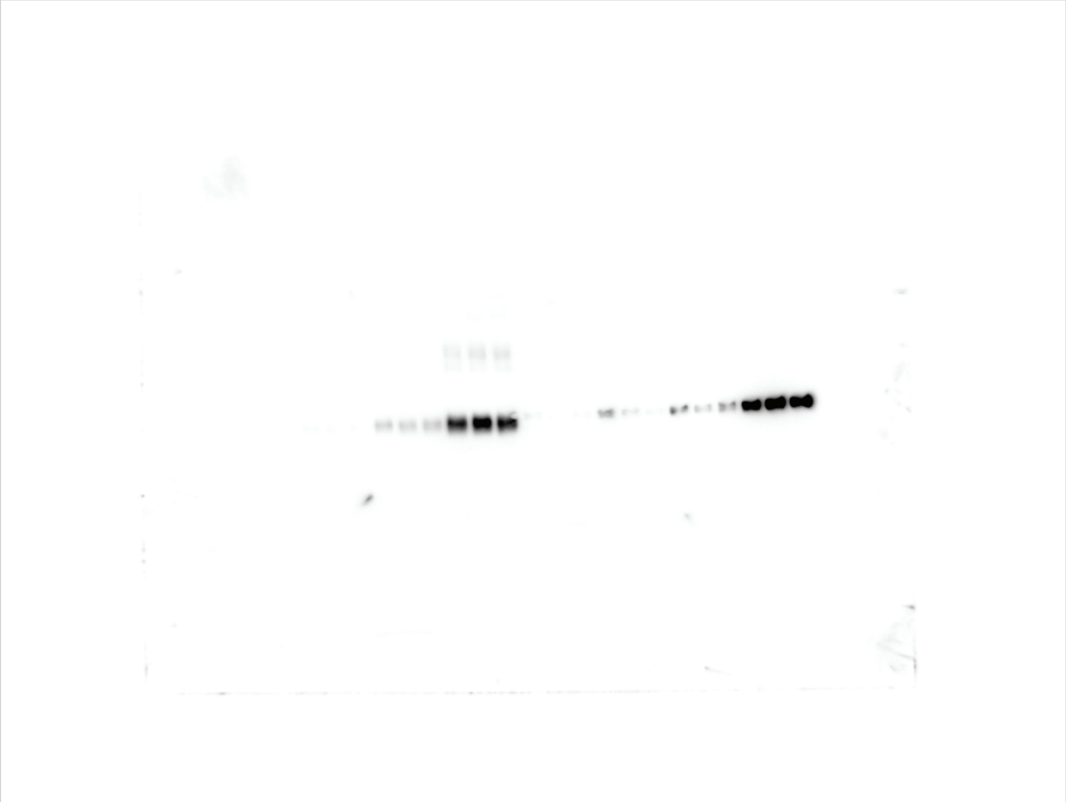

Source Data
Western Blot raw images with highlighted contrast
Figure 5C

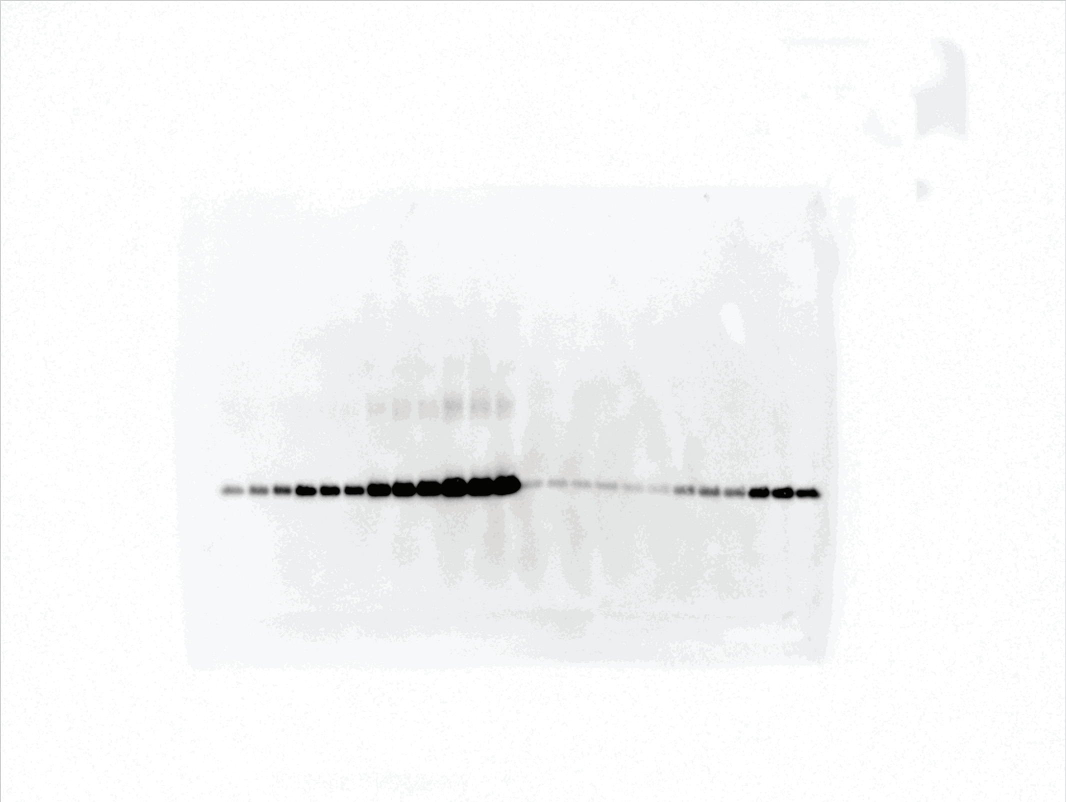

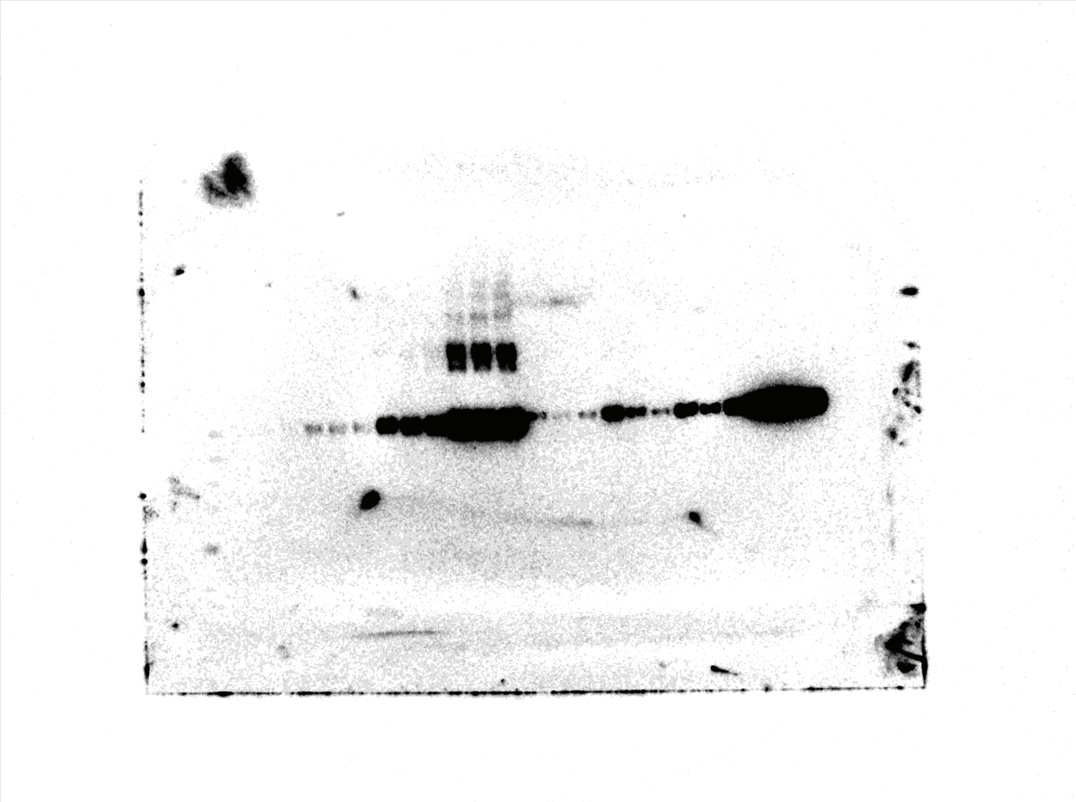

Supplement: Supplementary file 1 — Supplementary Information 1. [file 41598_2023_31900_MOESM1_ESM.pdf]
